# Supplementary material for: Norfluoxetine inhibits TREK-2 K2P channels by multiple mechanisms including state-independent effects on the selectivity filter gate
Source: J Gen Physiol. 2021 May 25;153(8):e202012812. doi: 10.1085/jgp.202012812 (PMC8155809; doi:10.1085/jgp.202012812)
Supplement: Table S1 — shows a comparison of single-channel parameters of single TREK-2 channels depicted in Fig. S2. [file JGP_202012812_TableS1.docx]

| **Mode** | **V (mV)** | **τ_O_ (ms)** | **τ_C1_ (ms)** | **A_C1_ (%)** | **τ_C2_ (ms)** | **A_C2_ (%)** | **τ_C3_ (ms)** | **A_C3_ (%)** | **τ_C4_ (ms)** | **A_C4_ (%)** | **τ_C5_ (ms)** | **A_C5_ (%)** |
| --- | --- | --- | --- | --- | --- | --- | --- | --- | --- | --- | --- | --- |
| **Standard** | **+60** | 1.2 | 0.17 | 45 | 0.88 | 20 | 6.9 | 16 | 24 | 18 | 145 | 1 |
| **High P_O_** | **+60** | 4.5 | 0.12 | 73 | 1.1 | 27 | - | - | - | - | - | - |
| **Standard** | **-60** | 0.24 | 0.042 | 24 | 0.11 | 58 | 0.4 | 7 | 23 | 2 | 140 | 8 |
| **High P_O_** | **-60** | 0.27 | 0.09 | 97 | 0.7 | 3 | - | - | - | - | - | - |

**Table S1. Comparison of single-channel parameters of single TREK-2 channels depicted in Supplementary Figure S2.** τ_O_, mean open time; τ_Ci_ and A_Ci_, mean lifetimes and corresponding areas of closed times (*i*=1-5).
